# Sleep Abnormalities in SLC13A5 Citrate Transporter Disorder

**DOI:** 10.3390/genes15101338

**Published:** 2024-10-18

**Authors:** Raegan M. Adams, Can Ozlu, Lauren E. Bailey, Rayann M. Solidum, Sydney Cooper, Carrie R. Best, Jennifer Elacio, Brian C. Kavanaugh, Tanya L. Brown, Kimberly Nye, Judy Liu, Brenda E. Porter, Kimberly Goodspeed, Rachel M. Bailey

**Affiliations:** 1 Center for Alzheimer’s and Neurodegenerative Diseases, UT Southwestern Medical Center, Dallas, TX 75390, USA; raegan.adams@utsouthwestern.edu (R.M.A.);; 2 Department of Pediatrics, UT Southwestern Medical Center, Dallas, TX 75390, USA; 3 Department of Neurology and Neurological Sciences, Stanford University, Palo Alto, CA 94304, USA; 4 Perot Neuroscience Translational Research Center, UT Southwestern Medical Center, Dallas, TX 75390, USA; 5 Department of Molecular Biology, Cell Biology and Biochemistry, Brown University, Providence, RI 02912, USA; 6 Department of Psychiatry & Human Behavior, Brown University, Providence, RI 02912, USA; 7 TESS Research Foundation, Menlo Park, CA 94026, USA; 8 Department of Neurology, Brown University, Providence, RI 02912, USA

**Keywords:** SLC13A5, sleep, SDSC, EEG, patients, mice

## Abstract

Background: SLC13A5 Citrate Transporter Disorder is a rare pediatric neurodevelopmental disorder. Patients have epilepsy, developmental disability, and impaired mobility. While sleep disorders are common in children with neurodevelopmental disorders, sleep abnormalities have not been reported in SLC13A5 patients. Methods: Here, we assessed sleep disturbances in patients through caregiver reported surveys and in a transgenic mouse model of SLC13A5 deficiency. A total of 26 patients were evaluated with the Sleep Disturbance Scale for Children three times over a one-year span. Sleep and wake activities were assessed in the SLC13A5 knock-out (KO) mice using wireless telemetry devices. Results: A high burden of clinically significant sleep disturbances were reported in the patients, with heterogeneous symptoms that remained stable across time. While sleep disturbances were common, less than 30% of patients were prescribed medications for sleep. Comparatively, in SLC13A5 KO mice using EEG recordings, significant alterations were found during light cycles, when rodents typically sleep. During the sleep period, SLC13A5 mice had increased activity, decreased paradoxical sleep, and changes in absolute power spectral density, indicating altered sleep architecture in the mouse model. Conclusions: Our results demonstrate a significant component of sleep disturbances in SLC13A5 patients and mice, highlighting a potential gap in patient care. Further investigation of sleep dysfunction and the underlying etiologies of sleep disturbances in SLC13A5 citrate transporter disorder is warranted.

## 1. Introduction

SLC13A5 Citrate Transporter Disorder (SLC13A5 Deficiency, SLC13A5 Epilepsy, Development, and Epileptic Encephalopathy DEE25, OMIM #615905) is a rare pediatric neurodevelopmental disorder characterized by the onset of seizures in the neonatal period, global developmental delay, limited verbal communication abilities, and motor impairments, including dystonia, hypotonia, and difficulty walking [1,2]. Nearly all patients present with onset of seizures within the first day of life; however, there is variability across the patients’ seizure burden and an overall reduction in seizure with age [3]. This disorder is caused by recessive, loss of function variants in the *SLC13A5* gene, which encodes a plasma membrane sodium citrate co-transporter (NaCT) [4,5]. While NaCT is highly expressed in both the brain and liver, the predominant phenotype is neurodevelopmental [6]. To date, there are more than 40 identified variants, which often cause reduced or complete loss of citrate transport into cells [2,7,8,9]. Deficiency in citrate transport results in excess extracellular citrate and numerous metabolic alterations, with changes observed in glutamate and GABA trafficking [10].

Current treatment options for SLC13A5 focus on controlling seizures with anti-seizure medications, therapies to support development, durable medical equipment for mobility, and medications or procedures to address other comorbidities, such as feeding issues. However, therapies to address the underlying genetic abnormality are not yet clinically available. There are many challenges to address when developing therapeutics for rare neuro–genetic conditions, including small patient population sizes and incomplete understanding of the pathophysiology and natural history of the disease. An ongoing natural history study for SLC13A5 is characterizing disease trajectories of one of the largest patient cohorts in a study to date. This natural history study is a high-priority research area identified by the TESS Research Foundation, the 501(c)3 non-profit organization focused on driving research for SLC13A5 Citrate Transporter Disorder [11]. This study also represents an active, patient-centered collaboration between three academic institutions and TESS Research Foundation. This is a two-year, multisite, longitudinal study with neuropsychological evaluations, motor assessments, electroencephalograms (EEGs), medication and seizure diaries, sleep evaluations, and quality of life assessments. There is an additional remote arm of this study to capture disease characteristics and trajectories from the participants who are unable to travel to the study sites. In addition to studying disease progression, the data collected will inform clinical care guidelines, as well as the design of future clinical trials.

Sleep disturbances are prevalent among children with neurodevelopmental and epileptic disorders [12,13,14,15]. Epilepsy and anti-seizure medications are associated with higher rates of sleep disturbances [16,17]. Furthermore, there is a well-documented correlation in epilepsy between sleep deprivation and an increased risk of seizures [18]. While sleep has not been studied in SLC13A5 Citrate Transporter Disorder, prior retrospective studies reported sleep symptoms, including sleep-related breathing disorders, indicating a need to better understand sleep problems [19].

Here, we report on sleep characteristics from the SLC13A5 natural history study and assessed EEG and sleep behavior in a mouse model of SLC13A5. We found significant behavioral sleep disturbances in both patients and mice lacking functional SLC13A5 and a shift in EEG power spectra in the SLC13A5 deficient mice.

## 2. Materials and Methods

### 2.1. Recruitment and Consent

Participants with genetically and clinically confirmed SLC13A5 Citrate Transporter Disorder were identified via the TESS Research Foundation and offered enrollment in one of two SLC13A5 Natural History Studies. Patients living outside the US (NCT04681781) were offered a remote-only study, while those living within the US were offered either an in-person and remote (NCT06144957) study. The Institutional Review Board at Stanford approved the international portion of the Natural History Study, Protocol SLC13A5 Deficiency: A Prospective Natural History Study (International) 57342.

Under three separate protocols, at each study site, the in-person Natural History Study was approved by the following: the Institutional Review Board at Stanford (Protocol SLC13A5 Deficiency: A Prospective Natural History Study #57902); Brown University (#403420); and the University of Texas Southwestern (#STU-2020-0635). Prior to consent, the caregivers reviewed the protocol and discussed any concerns with the principal investigator and the study coordinators. Assent and a consent were provided via RedCap for the international study, and a paper consent was provided for the in-person study.

The studies spanned two years, with four time points at which sleep data were collected via the Sleep Disturbances Scale for Children: baseline, 6 months, 12 months, and 24 months. Patients were enrolled on a rolling basis, with some patients not having completed their 24 months visits at the time of this submission.

### 2.2. Sleep Disturbances Scale for Children (SDSC)

The Sleep Disturbances Scale for Children (SDSC) was developed in 1996 and is widely used to assess sleep problems in children [20]. The questionnaire includes 26 items, completed by parents or caregivers. The two initial questions establish how many hours per night the child sleeps and how long it takes for the child to fall asleep. The following 24 questions ask about the quality of sleep and behaviors during sleep, scored using a Likert scale of 1–5, ranging from never to daily, with a higher score representing a more severe sleep disturbance. These numbers are tallied into sub-scores, divided into six categories, which include disorders of initiating and maintaining sleep (DIMS), sleep breathing disorders (SBDs), disorders of arousal (DA), sleep–wake transition disorders (SWTDs), disorders of excessive somnolence (DoES), and sleep hyperhidrosis (SH). A total T score was calculated using a weighted sum of sub-scores.

The SDSC was originally developed to assess sleep disturbances specifically in children aged 6–16. While the patient population in the current study ranges from 5 months to 30 years (Table 1), the test has been used in younger children, as young as 6 months, with success in many cases using slightly altered versions [21,22,23,24]. Additionally, based on the severe developmental delay of patients, the SDSC was felt to be the overall most helpful tool to evaluate sleep disturbances of this population.

### 2.3. Animals

The mouse experiments were performed with approval from the Institutional Animal Care and Use Committee at University of Texas Southwestern (UTSW) Medical Center. SLC13A5 knock-out (KO) mice were originally obtained from Dr. Rafael de Cabo and maintained on a C57BL/6J background [25]. Both males and females were used in these studies and wildtype (WT) littermates were used as controls for comparison to KO mice. Mice were maintained under standard 12 h light and 12 h dark conditions and provided food and water ad libitum. As a part of an unrelated study, mice had been injected with 5 µL of sterile vehicle (5% D-sorbitol, 350 mM NaCl in phosphate buffered saline) via intrathecal lumbar puncture at post-natal day 10.

### 2.4. Telemetry Devices and Recordings

Three-month-old mice were implanted subcutaneously with an HD-X02 Data Systems International (DSI) telemetry probe (St. Paul, MN, USA), as previously described [26]. A dual channel transmitter was used for EEG recordings with coordinates at LH: AP +1.0, ML −1.5; and RH: AP −2.0, ML +2.0, as well as for EMG lead placement in the trapezius muscles. The recordings started approximately 10 days following surgery and were acquired over two 60 h sessions (three dark cycles and two light cycles), approximately two weeks apart. All EEG recordings were collected in the subject animal’s home cage in a temperature-controlled testing room maintained on a 12:12 h light–dark cycle. Animals were singly housed after EEG implantation to avoid possible device displacement due to cage-mate interactions.

### 2.5. EEG and EMG Data Analysis

Ponemah (Data Systems International, St. Paul, MN, USA, v6.51) was used to acquire data, and Neuroscore (Data Systems International, St. Paul, MN, USA, v.3.4.0) was used to analyze data. Activity counts were calculated by Neuroscore software and are based on the implant movement in relation to the PhysioTel receiver (Data Systems International, St. Paul, MN, USA) that each cage is placed on during telemetry data acquisition. The activity counts were separated into activity during the dark cycles and activity during the light cycles, summed for two (light) or three (dark) cycles per recording period and averaged between both recording periods for each animal. Sleep stages were categorized into active wake, quiet wake, slow-wave sleep, and paradoxical sleep (also known as REM sleep), and were automatically scored using the integrated Rodent Sleep Scoring program using a combination of delta and theta power, muscle tone, and movement in ten second epochs. The percentage of time spent in each stage was averaged between the two recording periods. Absolute power was automatically determined by the software into four frequency bands: delta (0.5–4 Hz), theta (4–8 Hz), alpha (8–12 Hz), and beta (12–30 Hz). Absolute power was quantified during the light and dark cycles based on recording time (light: 6:00 AM–6:00 PM, dark 6:00 PM–6:00 AM). A sleep stage based on Rodent Sleep Scoring was assigned and quantified for ten-second duration epochs and then averaged across both recording sessions.

### 2.6. Statistical Analysis

Based on previous publications using the SDSC in a diverse cohort that maximized sensitivity and specificity, an overall T score ≥ 56 or a subdomain score of ≥70 was considered a clinically significant sleep disturbance [21,27]. The SDSC data were presented as average ± standard deviation unless otherwise specified and were analyzed and graphed using GraphPad Prism Software (Boston, MA, USA, v10.2.0). For time course clinical studies, ordinary one-way ANOVA was used, followed by Dunnett’s multiple comparison test. For mouse studies, group sizes were verified using power analysis with a type 1 error of 5% and a power of 80% to detect an effect size greater than 0.8, which would suggest a large change (G*Power, Düsseldorf, Germany, v3.9.1.7). Sex differences were not found for general activity or power band spectra, so male and female data were collapsed to increase power. Sex was included as a variable for wake/sleep stages, as it was significantly impacted for three of the four stages. Data sets comparing one variable were analyzed using an unpaired Student’s *t*-test. Data sets comparing multiple variables were analyzed via two-way ANOVA, followed by Sidak’s multiple comparisons test. Effect size was assessed using eta squared (η^2^), where the interpretation of the effect size was as follows: 0.01 ≤ 0.05 (low effect), 0.06 ≤ 0.13 (moderate effect), and ≥0.14 (high effect). A value of *p* < 0.05 was considered significant for all statistical analyses.

## 3. Results

### 3.1. Participant Characteristics

The SLC13A5 natural history study comprised of 26 children and young adult participants with a mean age of 9.9 ± 8.1 years (the age range was from 5 months to 30 years old) at the time of enrollment. A total of 12 participants were from the United States and completed in-person visits, while 14 international participants had visits remotely. A total of 15 participants were male, and 11 were female (Table 1). At the time of preparation of this report, 76.9% (20/26) had completed the 6-month survey, 57.7% (15/26) had completed the 12-month survey, and 11.5% (3/26) had completed the 24-month survey. Only data from the 0-, 6-, and 12-month time points are presented. The questionnaire completion rates, sex ratios, and prevalence of sleep aids at study baseline for the in-person and remote cohorts were similar.

### 3.2. Overall Sleep Scores

**Figure 1:**
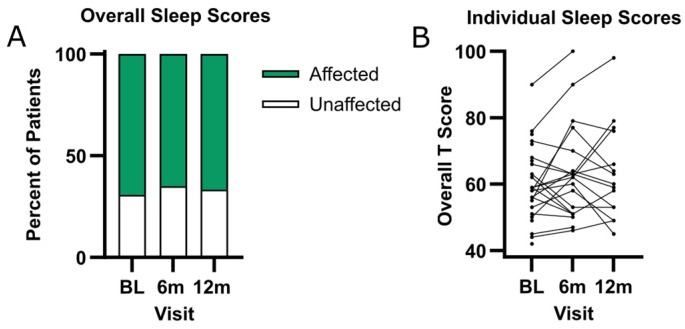
Sleep disturbances are prevalent in SLC13A5 patients. (**A**) Percentage of patients that had clinically significant (T score ≥ 56, shown in green) and non-clinically significant (T score < 56, shown in white) sleep disturbances, as assessed by the overall sleeping score from the SDSC over a one-year span. (**B**) Individual T scores for each visit. BL = baseline.

At baseline, 69.2% of participants (18/26) had clinically significant Sleep Disturbances Scale (SDSC) scores (overall T score ≥ 56), indicating that most patients had some component of sleep disturbance. The T scores ranged from 42 to 90, with a mean of 60.3 ± 11.1, indicating a wide range of sleep abnormalities among the participants (Figure 1A). The SDSC scores were mostly stable across the three time points during the first year of this study: 65.0% (13/20) of patients had a positive score (T score ≥ 56) at six months, and 66.7% (10/15) of patients had a positive score at 12 months. A proportion of 23.1% (6/26) of patients were taking medications for the purpose of sleep at the time of enrollment (Table 1). Medications included melatonin (*n* = 4) and gabapentin *(n* = 2). The overall T scores did not demonstrate statistically significant changes over time (Figure 1B). For the 15 patients that completed the first three visits, the mean difference from baseline to the final visit was 3.9 ± 10.6. Additionally, there was no correlation between age and baseline T scores; the T score averages did not differ between the in-person and remote cohorts or genders (Supplemental Figure S1).

### 3.3. Sleep Disorder Sub-Scores

**Figure 2:**
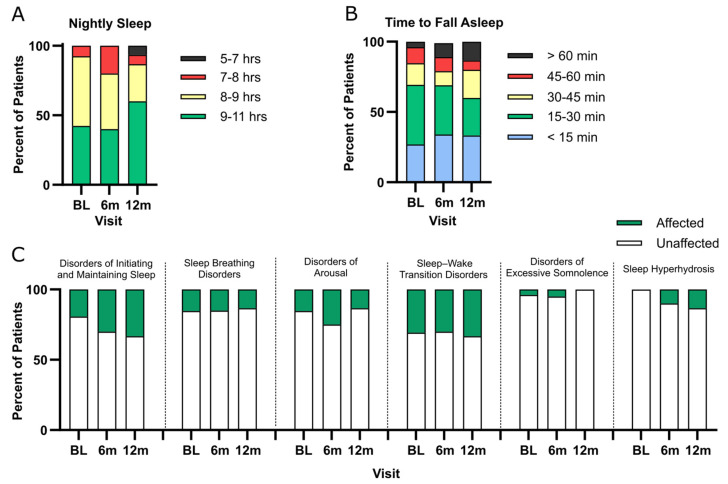
Patients had difficulties with sleep transitions and insomnia. (**A**) Nightly time spent sleeping and (**B**) time to fall asleep for patients. (**C**) Percentage of patients with a clinically significant sleep disorder. Affected = T score ≥ 70; unaffected = T score ≥ 70. BL = baseline.

There were no significant changes over visits in sleep duration or time to fall asleep, suggesting that patients’ sleep disturbances are generally stable over a one year period. At baseline, nearly half of all participants slept 9–11 h (42.3%, 11/26), half of patients slept 8–9 h (50.0%, 13/26), 7.7% (2/26) reported 7–8 h of sleep and no patients slept 5–6 h (Figure 2A). Similar trends and stability over time were seen for the time it took to fall asleep. At baseline, most patients required 15–30 min to fall asleep after going to bed (42.3%, 11/26), 26.9% (7/26) needed less than 15 min, 15.4% (4/26) needed 30–45 min, 11.6% (3/26) needed 45–60 min, and 3.8% (1/26) needed more than an hour (Figure 2B).

Sleep–wake transition disorders (8/26, 60.9 ± 14.4) and disorders of initiating and maintaining sleep (5/26, 64.5 ± 12.6) had the highest percentage of clinically significant subdomain scores for patients. Both sleep breathing disorders (55.5 ± 9.5) and disorders of arousal (52.3 ± 11.5) had four clinically significant patients each. Disorders of excessive somnolence (1/26, 50.5 ± 9.8) and sleep hyperhidrosis (0/26, mean score 48.1 ± 8.5) had the lowest percentage of positive scores (Figure 2C). Patients had trouble falling asleep, which is supported by the large range of time it took them to fall asleep (Figure 2B). A proportion of 73.1% (19/26) of patients were positive (T score ≥ 70) for a single sleep issue at baseline, and 26.3% (5/19) had difficulty with more than one sleep domain. There were no significant changes over visits in subdomain scores, suggesting that patients’ sleep disturbance subscales are generally stable over a one-year period.

### 3.4. Abnormal Sleep Structure in SLC13A5 KO Mice

**Figure 3:**
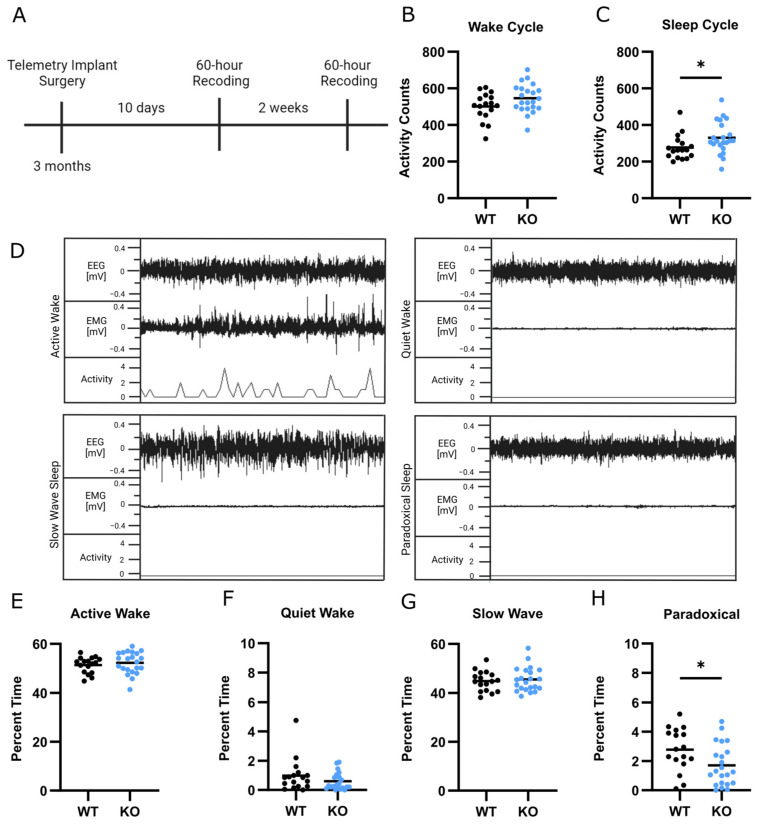
KO mice had decreased paradoxical sleep. (**A**) Timeline overview of EEG recordings. (**B**,**C**) Average EMG activity of WT and KO mice during wake cycles (**B**) and sleep cycles (**C**). Student’s *t*-test, * *p* < 0.05. (**D**) Representative EEG, EMG, and activity traces within the four stages of sleep of a WT animal. Each recording window represents one minute. (**E**–**H**) Percentage of time spent in active wake (**E**), quiet wake (**F**), slow-wave sleep (**G**), and paradoxical sleep (**H**). *n* = 17 WT, *n* = 22 KO. Two-way ANOVA genotype effect shown, * *p* < 0.05.

Having identified high rates of sleep initiation and waking abnormalities in patients with SLC13A5 Citrate Transporter Disorder, we next investigated whether the SLC13A5 mouse models exhibited these sleep complaints. WT and KO littermates were implanted with a wireless telemetry probe to measure general, EEG, and EMG activity over two 60 h recording periods (Figure 3A). During the dark cycles, when nocturnal mice are typically awake and active, the WT and KO mice had similar activity levels (Figure 3B). In contrast, the KO mice had a significant, moderate increase in activity compared to the WT mice during the light cycles when mice typically sleep, having on average of 330.0 activity counts, compared to 277.3 activity counts, per recording period (Figure 3C); (*t* test, t = 2.058, df = 35, *p* = 0.0471, η^2^ = 0.11). This suggests that KO mice may sleep less than WT mice. There were no differences in activity levels between males and females.

To assess the sleep architecture of WT and KO mice, we used EEG and EMG data to characterize four sleep/wake stages: active wake, quiet wake, slow-wave sleep, and paradoxical sleep (Figure 3D). We found that KO mice spent a similar percentage of time within active wake, quiet wake, and slow-wave sleep states compared to WT littermates, although these states were different between male and female mice (Figure 3E–G and Supplemental Table S1). Interestingly, paradoxical sleep was highly effected, with KO mice spending significantly less time in paradoxical sleep (Figure 3H); two-way ANOVA genotype effect (F (1, 35) = 5.840, *p* = 0.210, η^2^ = 0.14). Paradoxical sleep was unaffected by sex (Supplemental Table S1). This supports the idea that KO mice have altered sleep physiology, with paradoxical sleep being the most affected.

### 3.5. Decreased Power Spectra during Sleep in SLC13A5 KO Mice

**Figure 4:**
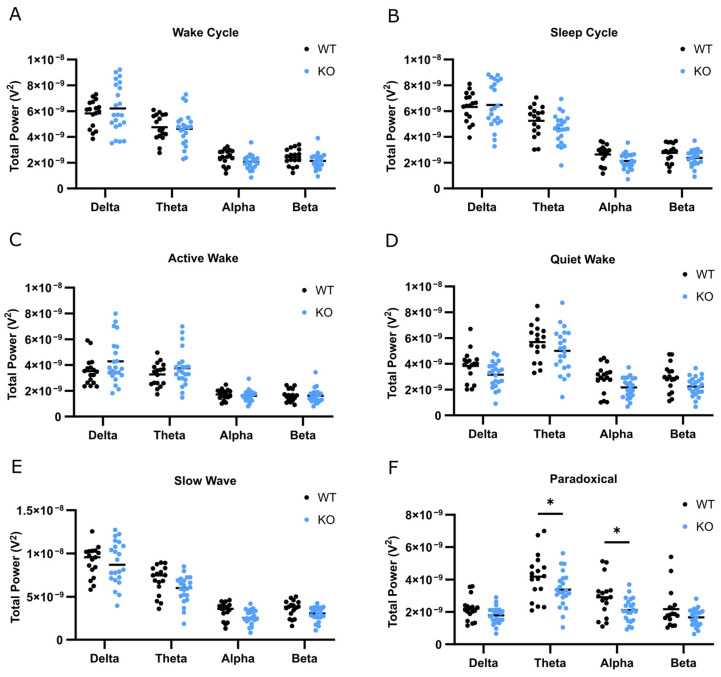
KO mice had altered power spectral densities during sleep. (**A**,**B**) Power spectral density (PSD) averaged over two, 60 h recordings during wake cycles (6 PM–6 AM) (**A**) and sleep cycles (6 AM–6 PM) (**B**). (**C**–**F**). PSD during active wake (**C**), quiet wake (**D**), slow-wave sleep (**E**), and paradoxical sleep (**F**), divided into delta, theta, alpha, and beta frequencies. *n* = 17 WT, *n* = 22 KO. Two-way ANOVA with Sidak’s multiple comparisons test, * *p* < 0.05.

We then performed power spectral density (PSD) analysis across 0–30 Hz frequencies. Frequency bands were defined as delta (0.5–4 Hz), theta (4–8 Hz), alpha (8–12 Hz), and beta (12–30 Hz). Within the dark cycle, absolute PSD was similar between genotypes (Figure 4A). During the light cycle though, there were small effects on the overall power spectra of KO mice as compared to WT mice (Figure 4B, two-way ANOVA genotype effect (F (1, 148) = 3.975, *p* = 0.0480, η^2^ = 0.01)). There was a trend of decreased theta and alpha waves in KO versus WT mice during the light cycle, although this difference was not statistically significant. We further examined power spectra within the specific sleep/wake stages. WT and KO mice had similar absolute power densities during the active wake stage (Figure 4C). In contrast, absolute spectral power was altered in KO mice relative to WT mice within quiet wake (two-way ANOVA genotype effect (F (1, 148) = 13.02, *p* = 0.0004, η^2^ = 0.04)), slow-wave sleep (two-way ANOVA genotype effect (F (1, 148) = 7.134, *p* = 0.0084, η^2^ = 0.01)), and paradoxical sleep stages (two-way ANOVA genotype effect (F (1, 148) = 16.09, *p* < 0.0001, η^2^ = 0.06)) (Figure 4D–F). In paradoxical sleep, theta (Sidak’s multiple comparisons test, *p* = 0.0418) and alpha power (Sidak’s multiple comparisons test, *p* = 0.0486) bands were significantly decreased in KO mice compared to WT mice. Power spectra density was similar between male and female mice. Together, these data suggest that aberrant brain activity is associated with decreased time spent in paradoxical sleep and that altered power spectra within sleep stages may underlie sleep disturbances in SLC13A5 KO mice.

## 4. Discussion

This study included one of the largest patient populations with SLC13A5 Citrate Transporter Disorder to date, and the ongoing natural history study is a high priority area of focus for the SLC13A5 Citrate Transporter Disorder community [11]. This collaboration across multiple academic institutions, as well as the TESS Research Foundation, demonstrates the utility of patient-centered research to advance the understanding of rare diseases such as SLC13A5 Citrate Transporter Disorder. This study is the first to characterize sleep disturbances in this group. These results showed that sleep disturbances were prevalent among the study participants, with 69.2% (18/26) of the surveyed patients having clinically significant scores for overall sleep disturbances and 73.1% (19/26) of patients having clinically significant scores for at least one sleep subcategory, as measured by the SDSC. Difficulties were prominent in falling and staying asleep, with the DIMS and SWTD having the highest sub-scores. Interestingly, 30.7% (8/26) of patients reported needing over 30 min to fall asleep. Patients remained stable in their sleep scores over the 12-month study period, suggesting that this is not a progressive or varied phenotype at the ages assessed.

Anti-seizure medications are known to affect sleep quality and structure [28]. SLC13A5 Citrate Transporter patients are typically prescribed a variety of one or more medications, with phenobarbital, valproic acid, and acetazolamide being the most common [29]. The effects of anti-seizure medications on sleep are variable and can induce both somnolence and insomnia events in patients. Because of the array of medications being taken, it is difficult to parse out the contributions of the underlying disorder versus medication effects. Of the 26 patients surveyed, only 23.1% (6/26) of patients were taking sleeping aids compared to the much larger number of patients reporting sleep disturbances, indicating a gap in patient care. Screening for sleep disturbances and treatment may help improve quality of life for SLC13A5 Citrate Transporter Disorder patients.

The SLC13A5 KO mouse model also has evidence of altered sleep physiology. Similar to patient phenotype presentation, there also seems to be a wide range of sleep disturbance within the mouse model. During the light cycles, when mice typically sleep, we found that the KO mice exhibited greater activity, which was associated with a decrease in overall PSD. The analysis of specific wake/sleep stages revealed that the KO mice spent significantly less time in paradoxical sleep compared to their WT littermates, while their time in the active wake stage was unchanged. Furthermore, power spectra were normal during active wake periods, but significantly altered in the KO mice during quiet wake, slow-wave sleep, and paradoxical sleep stages, with paradoxical sleep being the most affected. Paradoxical sleep, or REM sleep, is characterized by an EEG pattern similar to an awake state paired with a suppression of skeletal muscle activity [30]. Decreased REM sleep has been reported in several other developmental disorders, including Angelman’s Syndrome and Autism Spectrum Disorder [31,32]. Taken with the above patient data, results from SLC13A5 KO mice suggest that sleep disturbance is also a feature of SLC13A5 Citrate Transporter Disorder.

The theta and alpha EEG frequencies were the most significantly decreased in paradoxical sleep. Theta waves during paradoxical sleep play an integral roles in learning and memory formation, as well as in sleep transitioning [33,34]. Theta waves are implicated in the GABAergic synchronization of pyramidal cell activity [35,36], and a strong link exists between GABA and acetylcholine levels, with theta activity within the hippocampus, where a reduction in these neurotransmitters can lead to reduced theta power [37,38]. Similar to the patients, the SLC13A5 KO mice have metabolic perturbations and increased neuronal excitability [25,39,40]. To date, all diseases causing SLC13A5 result in reduced transport of extracellular citrate into cells. As citrate is needed for the synthesis of GABA and other neurotransmitters, altered neurotransmitter levels may be a potential mechanism contributing to sleep disturbances and altered power spectra in mice [41]. While alpha power is not traditionally considered to be integral in paradoxical sleep, alpha power is a hallmark of relaxed wakefulness [42]. Given the trending decrease in alpha power in quiet wake sleep, this could indicate the difficulty of the SLC13A5 KO mice in falling asleep, which is consistent with the observed patient data.

While our results highlight sleep disturbances in patients with SLC13A5 Citrate Transporter Disorder and in SLC13A5 KO mice, there are limitations to consider, and future studies are needed. The SDSC was chosen because of its historical use in the evaluation of sleep difficulties in children; however, not all questions may be applicable for this specific patient population. Other groups have successfully adapted the SDSC to fit their patient population, and this may be a worthwhile pursuit for SLC13A5. Another limitation is that at the point of publication, the patients had been followed for up to 24 months, which does not capture the full spectrum of developmental points for most patients. As such, longer assessments are needed in patients and should include evaluating sleep architecture. This would be insightful for understanding whether paradoxical sleep alterations detected in mice are mirrored in humans. Our mouse study focused on a single age-group; evaluating whether sleep disturbances in mice are dynamic over different developmental time points would be valuable in understanding disease stability and trajectory. The results from mice could complement findings from human studies and be used to identify clinical trial outcome measurements for therapeutic development.

## 5. Conclusions

In summary, our results demonstrate for the first time a significant component of sleep disturbance in SLC13A5 Citrate Transporter Disorder in both data from natural history study participants and a SLC13A5 KO mouse model. This is important, as sleep disturbances can negatively impact patient quality of life. The data herein may be helpful in the clinical setting, as well as for outcome selection considerations in future therapeutic trials, as novel therapies are under development for SLC13A5.

## Figures and Tables

**Table 1:** Demographics.

	In-Person Cohort	Remote Cohort
Age in Years (mean ± SD) Range	10.0 ± 5.7 3.3–18.5	9.8 ± 10.0 0.4–30.2
Gender (M, F)	7 M, 5 F	8 M, 6 F
	Baseline: 100% (12/12)	Baseline: 100% (14/14)
Visit Completion Rate	6 Months: 75.0% (9/12)	6 Months: 78.6% (11/14)
	12 Months: 58.3% (7/12)	12 Months: 57.1% (8/14)
Sleep Aids	16.7% (2/12)	28.6% (4/14)

## Data Availability

The raw data supporting the conclusions of this article will be made available by the authors upon reasonable request.

## Supplementary Materials

The following supporting information can be downloaded at: https://www.mdpi.com/article/10.3390/genes15101338/s1, Supplemental Figure S1: Baseline characteristics of participants. Supplemental Table S1: Summary of percentage of time spent in sleep stages measured in female and male WT and KO mice and analyzed via two-way ANOVA.

## References

[1] Thevenon J., Milh M., Feillet F., St-Onge J., Duffourd Y., Juge C., Roubertie A., Heron D., Mignot C., Raffo E. (2014). Mutations in SLC13A5 cause autosomal-recessive epileptic encephalopathy with seizure onset in the first days of life. Am. J. Hum. Genet..

[2] Goodspeed K., Liu J.S., Nye K.L., Prasad S., Sadhu C., Tavakkoli F., Bilder D.A., Minassian B.A., Bailey R.M. (2022). SLC13A5 Deficiency Disorder: From Genetics to Gene Therapy. Genes.

[3] Matricardi S., De Liso P., Freri E., Costa P., Castellotti B., Magri S., Gellera C., Granata T., Musante L., Lesca G. (2020). Neonatal developmental and epileptic encephalopathy due to autosomal recessive variants in SLC13A5 gene. Epilepsia.

[4] Beltran A.S. (2024). Novel Approaches to Studying SLC13A5 Disease. Metabolites.

[5] Gopal E., Miyauchi S., Martin P.M., Ananth S., Srinivas S.R., Smith S.B., Prasad P.D., Ganapathy V. (2007). Expression and functional features of NaCT, a sodium-coupled citrate transporter, in human and rat livers and cell lines. Am. J. Physiol. Gastrointest. Liver Physiol..

[6] Kumar A., Cordes T., Thalacker-Mercer A.E., Pajor A.M., Murphy A.N., Metallo C.M. (2021). NaCT/SLC13A5 facilitates citrate import and metabolism under nutrient-limited conditions. Cell Rep..

[7] Hardies K., de Kovel C.G., Weckhuysen S., Asselbergh B., Geuens T., Deconinck T., Azmi A., May P., Brilstra E., Becker F. (2015). Recessive mutations in SLC13A5 result in a loss of citrate transport and cause neonatal epilepsy, developmental delay and teeth hypoplasia. Brain.

[8] Pajor A.M., de Oliveira C.A., Song K., Huard K., Shanmugasundaram V., Erion D.M. (2016). Molecular Basis for Inhibition of the Na+/Citrate Transporter NaCT (SLC13A5) by Dicarboxylate Inhibitors. Mol. Pharmacol..

[9] Klotz J., Porter B.E., Colas C., Schlessinger A., Pajor A.M. (2016). Mutations in the Na(+)/citrate cotransporter NaCT (SLC13A5) in pediatric patients with epilepsy and developmental delay. Mol. Med..

[10] Bainbridge M.N., Cooney E., Miller M., Kennedy A.D., Wulff J.E., Donti T., Jhangiani S.N., Gibbs R.A., Elsea S.H., Porter B.E. (2017). Analyses of SLC13A5-epilepsy patients reveal perturbations of TCA cycle. Mol. Genet. Metab..

[11] Brown T.L., Bainbridge M.N., Zahn G., Nye K.L., Porter B.E. (2024). The growing research toolbox for SLC13A5 citrate transporter disorder: A rare disease with animal models, cell lines, an ongoing Natural History Study and an engaged patient advocacy organization. Ther. Adv. Rare Dis..

[12] Ho N.T., Kroner B., Grinspan Z., Fureman B., Farrell K., Zhang J., Buelow J., Hesdorffer D.C., Rare Epilepsy Network Steering Committee (2018). Comorbidities of Rare Epilepsies: Results from the Rare Epilepsy Network. J. Pediatr..

[13] Esbensen A.J., Schwichtenberg A.J. (2016). Sleep in Neurodevelopmental Disorders. Int. Rev. Res. Dev. Disabil..

[14] Cilliler A.E., Guven B. (2020). Sleep quality and related clinical features in patients with epilepsy: A preliminary report. Epilepsy Behav..

[15] Agar G., Brown C., Sutherland D., Coulborn S., Oliver C., Richards C. (2021). Sleep disorders in rare genetic syndromes: A meta-analysis of prevalence and profile. Mol. Autism.

[16] Xu L., Guo D., Liu Y.Y., Qiao D.D., Ye J.Y., Xue R. (2018). Juvenile myoclonic epilepsy and sleep. Epilepsy Behav..

[17] Lawthom C., Didelot A., Coppola A., Aledo-Serrano A., Fazekas B., Sainz-Fuertes R., Strzelczyk A. (2023). The impact of epilepsy and antiseizure medications on sleep: Findings from a large European survey in adults with epilepsy and matched controls. Epilepsy Behav..

[18] Dell’Aquila J.T., Soti V. (2022). Sleep deprivation: A risk for epileptic seizures. Sleep Sci..

[19] Brown T.L., Nye K.L., Porter B.E. (2021). Growth and Overall Health of Patients with SLC13A5 Citrate Transporter Disorder. Metabolites.

[20] Bruni O., Ottaviano S., Guidetti V., Romoli M., Innocenzi M., Cortesi F., Giannotti F. (1996). The Sleep Disturbance Scale for Children (SDSC). Construction and validation of an instrument to evaluate sleep disturbances in childhood and adolescence. J. Sleep Res..

[21] Romeo D.M., Bruni O., Brogna C., Ferri R., Galluccio C., De Clemente V., Di Jorio M., Quintiliani M., Ricci D., Mercuri E. (2013). Application of the sleep disturbance scale for children (SDSC) in preschool age. Eur. J. Paediatr. Neurol..

[22] Romeo D.M., Cordaro G., Macchione E., Venezia I., Brogna C., Mercuri E., Bruni O. (2021). Application of the Sleep Disturbance Scale for Children (SDSC) in infants and toddlers (6–36 months). Sleep Med..

[23] Lecuelle F., Gustin M.P., Leslie W., Mindell J.A., Franco P., Putois B. (2020). French validation of the sleep disturbance scale for children (SDSC) in young children (aged 6 months to 4 years). Sleep Med..

[24] Chen X., Xu P., Chen Y., Chen S., Yao Y., Lin X. (2022). Validation of the sleep disturbance scale for children (SDSC) in infants and toddlers from mainland China. Front. Psychiatry.

[25] Birkenfeld A.L., Lee H.Y., Guebre-Egziabher F., Alves T.C., Jurczak M.J., Jornayvaz F.R., Zhang D., Hsiao J.J., Martin-Montalvo A., Fischer-Rosinsky A. (2011). Deletion of the mammalian INDY homolog mimics aspects of dietary restriction and protects against adiposity and insulin resistance in mice. Cell Metab..

[26] Copping N.A., Silverman J.L. (2021). Abnormal electrophysiological phenotypes and sleep deficits in a mouse model of Angelman Syndrome. Mol. Autism.

[27] Moavero R., Voci A., Romigi A., Bisulli F., Luisi C., Matricardi S., La Briola F., Mazzone L., Valeriani M., Curatolo P. (2022). Questionnaire-based assessment of sleep disorders in an adult population of Tuberous Sclerosis Complex. Sleep Med..

[28] Liguori C., Toledo M., Kothare S. (2021). Effects of anti-seizure medications on sleep architecture and daytime sleepiness in patients with epilepsy: A literature review. Sleep Med. Rev..

[29] Yang Q.Z., Spelbrink E.M., Nye K.L., Hsu E.R., Porter B.E. (2020). Epilepsy and EEG Phenotype of SLC13A5 Citrate Transporter Disorder. Child Neurol. Open.

[30] Peever J., Fuller P.M. (2017). The Biology of REM Sleep. Curr. Biol..

[31] Miano S., Bruni O., Leuzzi V., Elia M., Verrillo E., Ferri R. (2004). Sleep polygraphy in Angelman syndrome. Clin. Neurophysiol..

[32] Buckley A.W., Rodriguez A.J., Jennison K., Buckley J., Thurm A., Sato S., Swedo S. (2010). Rapid eye movement sleep percentage in children with autism compared with children with developmental delay and typical development. Arch. Pediatr. Adolesc. Med..

[33] Hutchison I.C., Rathore S. (2015). The role of REM sleep theta activity in emotional memory. Front. Psychol..

[34] Adamantidis A.R., Gutierrez Herrera C., Gent T.C. (2019). Oscillating circuitries in the sleeping brain. Nat. Rev. Neurosci..

[35] Cobb S.R., Buhl E.H., Halasy K., Paulsen O., Somogyi P. (1995). Synchronization of neuronal activity in hippocampus by individual GABAergic interneurons. Nature.

[36] Soltesz I., Deschenes M. (1993). Low- and high-frequency membrane potential oscillations during theta activity in CA1 and CA3 pyramidal neurons of the rat hippocampus under ketamine-xylazine anesthesia. J. Neurophysiol..

[37] Yoder R.M., Pang K.C. (2005). Involvement of GABAergic and cholinergic medial septal neurons in hippocampal theta rhythm. Hippocampus.

[38] Li S., Topchiy I., Kocsis B. (2007). The effect of atropine administered in the medial septum or hippocampus on high- and low-frequency theta rhythms in the hippocampus of urethane anesthetized rats. Synapse.

[39] Henke C., Tollner K., van Dijk R.M., Miljanovic N., Cordes T., Twele F., Broer S., Ziesak V., Rohde M., Hauck S.M. (2020). Disruption of the sodium-dependent citrate transporter SLC13A5 in mice causes alterations in brain citrate levels and neuronal network excitability in the hippocampus. Neurobiol. Dis..

[40] Milosavljevic S., Glinton K.E., Li X., Medeiros C., Gillespie P., Seavitt J.R., Graham B.H., Elsea S.H. (2022). Untargeted Metabolomics of SLC13A5 Deficiency Reveal Critical Liver-Brain Axis for Lipid Homeostasis. Metabolites.

[41] Kopel J.J., Bhutia Y.D., Sivaprakasam S., Ganapathy V. (2021). Consequences of NaCT/SLC13A5/mINDY deficiency: Good versus evil, separated only by the blood-brain barrier. Biochem. J..

[42] Schwabedal J.T., Riedl M., Penzel T., Wessel N. (2016). Alpha-wave frequency characteristics in health and insomnia during sleep. J. Sleep Res..

