# Supplementary material for: Sleep Abnormalities in SLC13A5 Citrate Transporter Disorder
Source: Genes (Basel). 2024 Oct 18;15(10):1338. doi: 10.3390/genes15101338 (PMC11507356; doi:10.3390/genes15101338)
Supplement: Supplementary file 1 [file genes-15-01338-s001.zip › genes-3233679-supplementary.pdf]

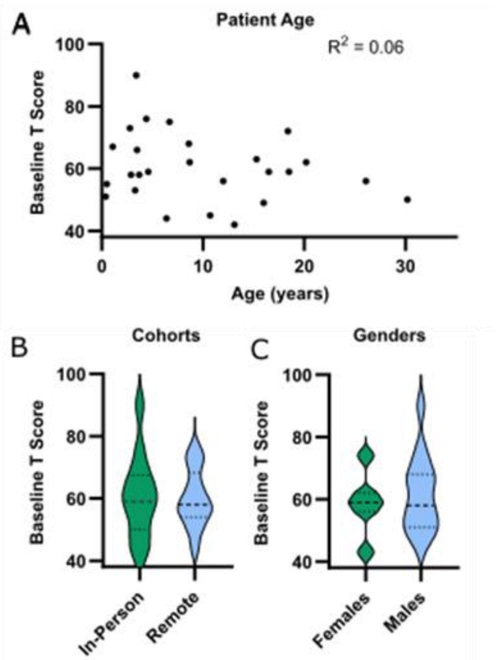

Supplemental Figure S1: Baseline characteristics of participants. **A.** Correlation between age of patients and baseline T score. **B.** Comparison of baseline T scores of domestic and international cohorts. C. Baseline T scores of female and male participants.

Supplemental Table S1: Summary of percent time spent in sleep stages measured from female and male WT and KO mice and analyzed via two-way ANOVA

Stage	Genotype		Sex		Interaction	
	**F(DFn, DFd)**	**P value**	**F(DFn, DFd)**	**P value**	**F(DFn, DFd)**	**P value**
Wake	F(1, 35) = 1.58	P=0.22	F(1, 35) = 49.7	p<0.0001	F(1, 35) = 2.62	P=0.11
Quiet Wake	F(1, 35) = 2.54	P=0.12	F(1, 35) = 7.18	p<0.05	F(1, 35) = 2.45	P=0.13
Slow wave	F(1, 35) = 0.20	P=0.66	F(1, 35) = 12.1	p<0.01	F(1, 35) = 2.77	P=0.11
Paradoxical	F(1, 35) = 5.84	p<0.05	F(1, 35) = 2.44	P=0.13	F(1, 35) = 0.75	P=0.39
